# Supplementary material for: Exploiting Potential Biotechnological Applications of Poly-γ-glutamic Acid Low Molecular Weight Fractions Obtained by Membrane-Based Ultra-Filtration
Source: Polymers (Basel). 2022 Mar 16;14(6):1190. doi: 10.3390/polym14061190 (PMC8949788; doi:10.3390/polym14061190)
Supplement: Supplementary file 1 [file polymers-14-01190-s001.zip › polymers-1591708-supplementary.pdf]

Supplementary Material

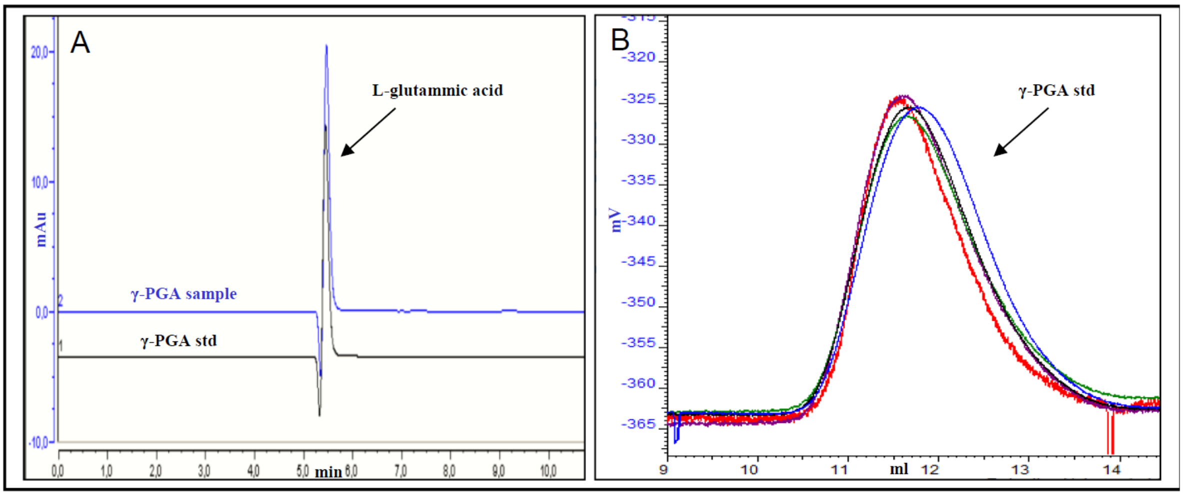

Figure S1. UHPLC chromatogram of a standard (std) sample of γ-PGA (black line), analysed on the base of its L-glutamic acid content after acidic hydrolysis of the polymer, in comparison with the chromatogram of a representative γ-PGA sample (blue line) from the membrane purification process (A). RI chromatograms of SEC-TDA analyses of the different γ-PGA standards used to determine the dn·dc^{-1}value (B).

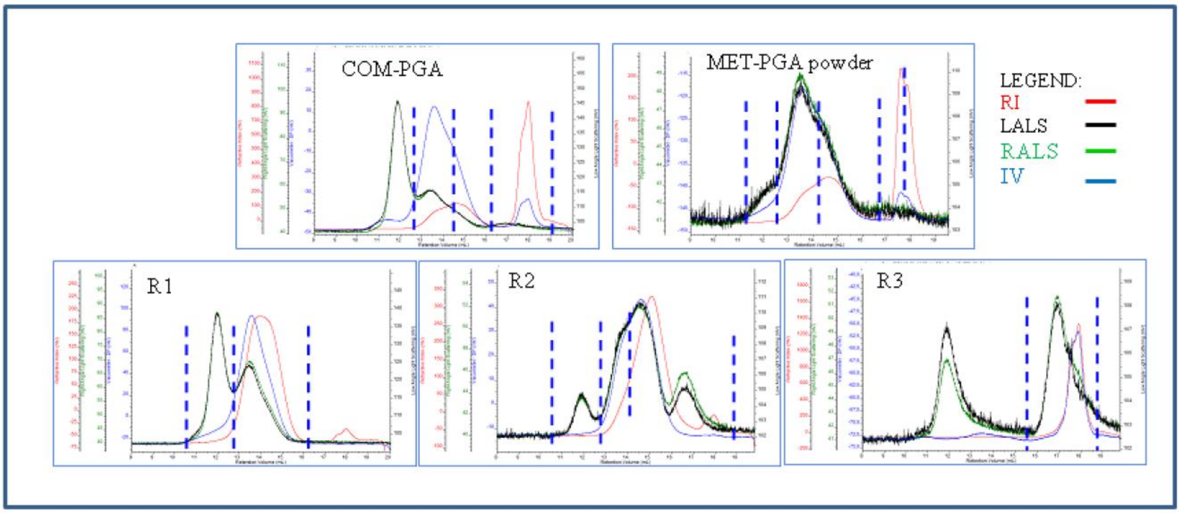

Figure S2. Complete SEC-TDA chromatograms of the different γ-PGA samples with the signals of RI (red line), of the viscosimeter (blue line) and of the right angle (green) and low angle light scattering (black line): Commercial γ-PGA , MET-PGA powder, 100 kDa retentate (R1), 3 kDa retentate (R2) and of the nano-filtration retentate (R3). The dot blue lines indicate how were integrated the different species.

S1. Thermal Analysis of MET-PGA, R1 and R2 Powder Based Films

From the analysis of MET-PGA powder thermograms (Figure S3A,B), it is possible to detect two different regions of weight loss. In the first one, from room temperature up to 150 °C, the sample loosed about 10–15% of its weight, and the weight loss was attributed to moisture and methanol evaporation. The second mass loss process was observed in the range of 200–400 °C and corresponded to the major stage of the thermal weight loss. This region was characterized by the decomposition of the different γ-PGA fractions, occurring in two different thermal degradation kinetics with a whole weight loss of about 60%. The first decomposition process was characterized by a peak of low intensity occurring at around 200 °C, likely due to the degradation of low Mw γ-PGA chain(s), less thermally stable. The second decomposition occurred at 300 °C and more intense was characterized by a neat and sharp peak attributed to higher Mw γ-PGA chain(s).

In particular, besides unbound and bound water evolution in the range of 50–200 °C, R1 powder sample evidenced only one peak of γ-PGA thermal decomposition at around 330 °C (Figures S3A,B). This outcome was

consistent with the sharp molecular weight distribution previously found by SEC-TDA analysis carried out after the ultra-filtration process with 100 kDa cut-off membrane. As far as the R2 powder (Figures S3A,B) and film samples (Figures 6A,B,D,E) are concerned, besides the two distinct kinetics related to unbound (80 °C) and freeze bound (180 °C) water removal, two different decomposition patterns of γ-PGA were detected. This result seems in agreement with the two different Mw γ-PGA fractions (52.7 and 17.9 kDa) found by SEC-TDA analysis, and it is very likely that γ-PGA with lower Mw degraded at a lower temperature.

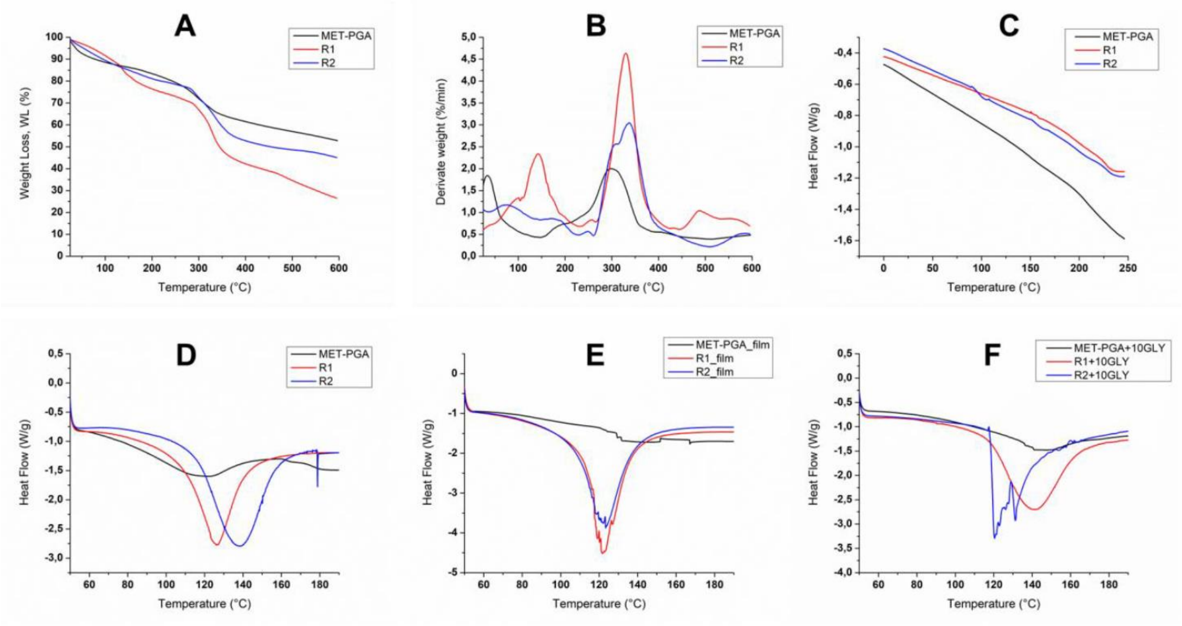

Figure S3. Thermogravimetric TGA (A), differential thermogravimetric DTG (B) and differential scanning calorimetric analysis DSC (second heating run C) of powder samples. DSC first heating run of powders (D), neat films (E) and glycerol doped films (F).

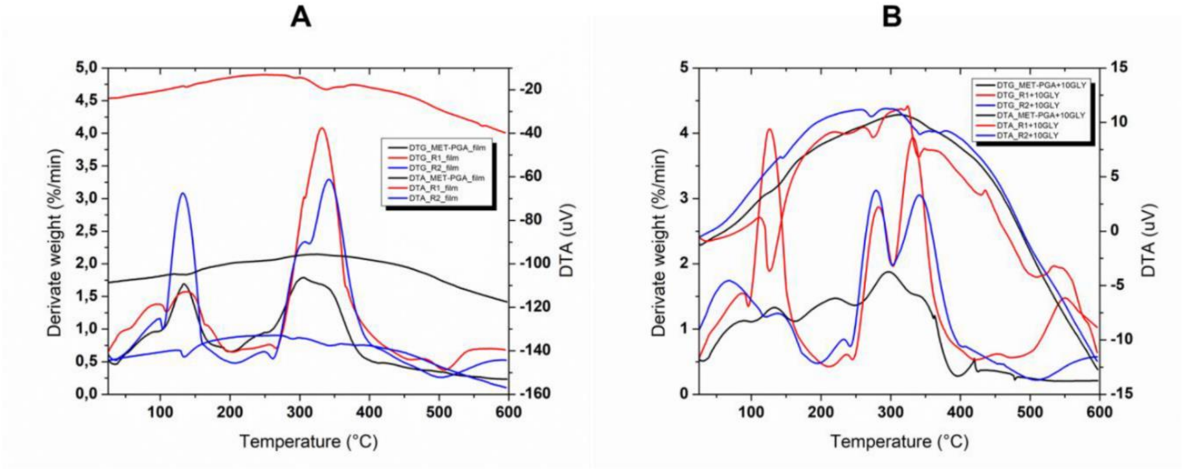

Figure S4. Derivative Thermogravimetry (DTG) and Differential Thermal Analysis (DTA) of MET-PGA, R1 and R2 neat films (A) and glycerol doped films (B).
